# Baseline dasabuvir resistance in Hepatitis C virus from the genotypes 1, 2 and 3 and modeling of the NS5B-dasabuvir complex by the *in silico* approach

**DOI:** 10.1080/20008686.2018.1528117

**Published:** 2018-10-05

**Authors:** Dario Akaberi, Assar Bergfors, Midori Kjellin, Nader Kameli, Louise Lidemalm, Bhavya Kolli, Robert W. Shafer, Navaneethan Palanisamy, Johan Lennerstrand

**Affiliations:** a Clinical Microbiology, Department of Medical Sciences, Uppsala University, Uppsala, Sweden; b Department of Medical Biochemistry and Microbiology, Zoonosis Science Center, Uppsala University, Uppsala, Sweden; c Department of Medical Microbiology, NUTRIM school of Nutrition and Translational Research in Metabolism, Maastricht University Medical Center, Maastricht, The Netherlands; d Department of Medicine, Division of Infectious Diseases, Stanford University, Stanford, CA, USA; e HBIGS, University of Heidelberg, Heidelberg, Germany; f Institute of Biology II, University of Freiburg, Freiburg, Germany

**Keywords:** Hepatitis C virus (HCV), dasabuvir, resistance, *in silico* docking, molecular dynamics (MD) simulation

## Abstract

**Background:** Current combination treatments with direct-acting antiviral agents (DAAs) can cure more than 95% of hepatitis C virus (HCV) infections. However, resistance-associated substitutions (RASs) may emerge and can also be present in treatment-naïve patients. **Methods, results and discussion:** In this study, a semi-pan-genotypic population sequencing method was developed and used to assess all NS5B amino acid variants between residue positions 310 and 564. Our method successfully sequenced more than 90% of genotype (GT) 1a, 1b, 2b and 3a samples. By using the population sequencing method with a cut-off of 20%, we found the dasabuvir RASs A553V and C445F to be a baseline polymorphism of GT 2b (8 out of 8) and GT 3a (18 out of 18) sequences, respectively. In GT 1a and 1b treatment-naïve subjects (n=25), no high-fold resistance polymorphism/RASs were identified. We further predicted dasabuvir’s binding pose with the NS5B polymerase using the *in silico* methods to elucidate the reasons associated with the resistance of clinically relevant RASs. Dasabuvir was docked at the palm-I site and was found to form hydrogen bonds with the residues S288, I447, Y448, N291 and D318. The RAS positions 316, 414, 448, 553 and 556 were found to constitute the dasabuvir binding pocket.

## Introduction

Hepatitis C virus (HCV) is an enveloped, positive-sense, single-stranded RNA virus belonging to the Hepacivirus genus of the *Flaviviridae* family [1]. HCV infection is one of the major reasons for chronic liver diseases and if not treated on time, often leads to liver cirrhosis and hepatocellular carcinoma. It is estimated that approximately 71 million people are chronically infected with HCV worldwide [2]. HCV is classified into 7 genotypes (GTs), the most common GT globally being GTs 1 and 3 [3]. Furthermore, these GTs are further classified into subtypes such as a, b, c .etc representing nuances within specific GTs [3]. In Europe including Sweden, like globally, GTs 1 and 3 are more prevalent compared to other GTs [4,5].

Until 2011, treatment for chronic HCV infections constituted pegylated interferon alpha (pegIFN-α) and ribavirin combination, given for 24 or 48 weeks. The success rates for curing the patients were approximately 80% for GTs 2 and 3, and 40 – 50% for GT 1 [6]. However, after the advent of direct-acting antivirals (DAAs), a successful cure can be accomplished at as high rates as 95 – 99% for some GTs and drug combinations. The DAAs can be classified into NS3/protease inhibitors (PIs), NS5A inhibitors, NS5B nucleoside analog inhibitors (NI) and NS5B non-nucleoside analog inhibitors (NNI) [7]. The NNIs bind to sites outside of the active site and changes the conformation of the NS5B protein by an allosteric mechanism. The structure of HCV NS5B polymerase enables four different binding sites for the NNIs, two of these binding sites are in the thumb region while two other sites are in the palm region [8]. Resistance against NNIs occurs more frequently than NI because of the less conserved regions surrounding the NNI binding sites. Currently, only dasabuvir (i.e. under NS5B NNI class) has been approved and marketed to treat HCV GT 1 infections [9,10]. It binds to one of the two NNI binding sites in the palm region. Dasabuvir, marketed alone as Exviera in Europe, has been used in combination with Viekirax which contains the protease inhibitor paritaprevir and the NS5A inhibitor ombitasvir. In the US, the combination of dasabuvir, paritaprevir and ombitasvir is marketed as Viekira Pak [11–13].

Poor fidelity of the viral RNA dependent RNA polymerase (RdRP) and a high virion production in HCV are the main reasons for the development of resistance when using NNIs [14]. Additionally, suboptimal treatment regimens and non-compliance accelerate the mutation rates [15–17]. The emergence of resistance mutations in NS5B can occur in two ways; by a transition or by a transversion substitution. In general, a transition substitution has a lower energy and evolutionary barrier, and therefore occur more frequently than a transversion substitution [18,19]. The resistance-associated substitutions (RASs) against dasabuvir have been extensively characterized in the Kati et al study [20]. This study reported that dasabuvir is primarily active against GTs 1a and 1b. The predominant dasabuvir NS5B RASs in GT 1a viruses are C316Y and S556G. In GT 1b, the predominant RASs are C316Y and M414T. Studies have also reported treatment failure cases associated with the RASs C316N, M414T, A553T, G554S, S556R, S556G, G558R, D559G/N and Y561H in GT 1a viruses and C316Y/N, M414I and S556G in GT 1b viruses [11,21–23].

The aim of this present study was to develop and evaluate a semi-pan-genotypic population sequencing method to identify NNI-associated RASs in 54 treatment-naïve patients harboring GT 1a, 1b, 2b and 3a viruses. Additionally, we applied the *in silico* methods to study the resistance mechanism of these RASs. The detection of RASs with population sequencing method could be important to optimize the treatment of HCV infections.

## Material and methods

### Patient samples

Serum samples collected from 54 patients earlier were retrieved from the biobanks at Clinical Microbiology, Uppsala University hospital (ethical approval Dnr 2009/023). All samples came from patients with a known on-going HCV infection and had previously been genotyped. The patients were treatment naïve to NS5B inhibitors and also to other DAAs.

### Primer design

Degenerate primers given in Table 1 were designed using sequence data from the NCBI database. The accession numbers for reference GTs 1a, 1b, 2b, 3a and 4a used were AF011751, FJ390398, AY232749, JN714194 and Y11604, respectively. A total of 47 sequences were aligned and primer sites were picked from the NS5B region. Primer design was performed using OligoAnalyzer version 3.1 (https://eu.idtdna.com/calc/analyzer).

**Table 1.** List of primers used in this study for sequencing of HCV NS5B region by the population sequencing method.

Primers	Sequence 5´ to 3´	Position in HCV genome (H77)
1^st^ forward	TATGAYACCCGCTGYTTYGA	8256–8275
1^st^ reverse	GGGCAYGHGACABGCTGTGA	9303–9282
2^nd^ forward	ACCCGCTGYTTYGACTCVAC	8262–8281
2^nd^ reverse	GACASGCTGWGATADATGTC	9295–9276
Seq forward	ACGGAGGCTATGACYAGGTA	8619–8638
Seq reverse	CAGGARTTRACWGGRGTGTG	8824–8805

### RNA extraction, cDNA synthesis and nested PCR

RNA was extracted from the serum samples by using the bioMérieux’s NucliSENS® easyMAG® system and cDNA from RNA was synthesized by using the Invitrogen™ SuperScript™ III reverse transcriptase. Applied Biosystems’s GeneAmp® PCR system 9700 was used for synthesizing cDNA from RNA. The reverse transcription conditions used were as follows: 1 cycle at 25°C for 1 min, 1 cycle at 42°C for 60 min, 1 cycle at 85°C for 5 min and finally hold at 4°C.

Nested PCR was done using the Applied Biosystems® TaqMan® Universal PCR Master Mix. The NS5B primers used for the first round and the second round nested PCRs are given in Table 1. For the first round PCR, the reaction mixture consisted of 12.5 μL of the TaqMan® Master Mix (2X), 0.4 μM 1^st^ forward and 1^st^ reverse primers each and finally 3 μL of the cDNA with a total volume of 25 μL. The PCR program consisted of 1 cycle at 94°C for 4 min, 35 cycles (94°C for 30 sec, 50°C for 30 sec, and 72°C for 1.5 min), then a final extension at 72°C for 5 min and hold at 4°C. For the second (nested) PCR, 2 μL of the first round PCR product was used. The other components (including the concentrations) were the same as the first round PCR except here we used 2^nd^ forward and 2^nd^ reverse primers. Likewise, the PCR program was also the same except here the annealing was done at 55°C. The amplified products were later verified on 1.5% agarose gel. QIAquick® PCR Purification Kit from QIAGEN was used, according to the manufacturer’s guidelines, to clean and purify all the positive samples.

### Population sequencing and mutation analysis

Sanger sequencing was done using primers 2^nd^ forward and 2^nd^ reverse and Seq forward and Seq reverse as given in Table 1. Sequences were analyzed for the mutation(s) using the SeqScape Software v2.6 from Applied Biosystems® with the NS5B of HCV GT 1a strain H77 (accession number: AF011751) as a reference. Clinically relevant variants between the residues 310 and 564 were inspected and compared to known clinically relevant NS5B NNI class RASs put forth by the HCV drug development advisory group [7,24].

### In silico docking of dasabuvir with HCV NS5B polymerase and molecular dynamics (MD) simulations

The three-dimensional structure of dasabuvir was generated with the compound’s SMILES (PubChem ID: 56640146) using MolConverter v16.7.4.0 (by ChemAxon). The HCV NS5B polymerase (GT 1b, isolate BK) crystal structure with PDB ID: 4MKB [25] was retrieved from the protein data bank (https://www.rcsb.org/pdb/home/home.do). It should be noted that the NS5B sequence from the GT 1b isolate BK already possesses C316N substitution.

The NS5B crystal structure was prepared for docking (i.e. removal of solvents and ligands) using AutoDockTools v1.5.6. AutoDock Vina [26] and iGEMDOCK [27] programs were used for the *in silico* docking studies. While running the AutoDock Vina, the exhaustiveness was set to ‘80’ while the number of output poses was set to ‘20’. The search space for docking was defined by a box of size 30 x 30 x 30 (in Å^3^) with coordinates X = −9.054, Y = 8.528 and Z = 8.545. For the iGEMDOCK, the population size, the generations and the number of solutions were set to 1000, 150 and 50, respectively. The search space was automatically assigned by iGEMDOCK from the provided NS5B crystal structure bound to the ‘28V’ ligand. From the docking data, a consensus binding pose predicted by both the programs was selected and used for further MD simulations.

MD simulations were performed using GROMACS version 5.1.1 (http://www.gromacs.org/) [28]. The simulations were run on ‘Rackham’ cluster composed of 334 nodes for a total of 6080 CPUs. The Swedish National Infrastructure (SNIC) provided the advanced computational resources required for the MD simulations through the Uppsala Multidisciplinary Center for Advanced Computational Science (UPPMAX), under the project SNIC 2017/1–213. The HCV NS5B-dasabuvir system was prepared as follows: the AMBER99SB-ILDN force field parameters [29] were assigned to the NS5B RNA-dependent RNA polymerase using GROMACS, while general AMBER force field (GAFF) [30] parameters were assigned to dasabuvir using ANTECHAMBER [31]. Dasabuvir topologies compatible with GROMACS were then generated with ACPYPE [32]. The system was centered in a dodecahedron simulation box filled with TIP3P water while keeping a solute box distance of 1 nm. Sufficient Na^+^ and Cl^−^ ions were also added to neutralize the system and have a final NaCl concentration of 150 mM. Each system was energy minimized and equilibrated first under NVT ensemble for 100 ps to a final temperature of 300 K using the V-rescale thermostat [33], followed by NPT ensemble for 500 ps using the Parrinello-Rahman barostat [34] to a final pressure value of 1 bar. MD simulations of 40 ns were performed with an integration time of 2 fs. During the simulations, all bonds were constrained using the LINCS algorithm [35]. The VERLET cut-off scheme [36] was used with a cut-off of 1.0 nm for the calculation of short-range coulomb and van der Waals interactions, while long-range electrostatic interactions were computed with the PME algorithm [37,38]. Four simulation replicates were performed with randomly generated new starting velocities for the systems, according to Maxwell distribution at 300 K. The resulting molecular dynamics simulations trajectories were analyzed using GROMACS built-in tools.

## Results

### Distribution of GTs, nested PCR and RASs identified by the population sequencing method

The 54 samples in our study had the following GT distribution: 1a = 21 (38.9%), 1b = 5 (9.3%), 2b = 9 (16.7%) and 3a = 19 (35.1%). Nested PCR was performed on these samples and we found that for GT 1a 20 out of 21 (95%) samples were amplified successfully, for GT 1b 5 out of 5 (100%) samples were amplified successfully, for GT 2b 8 out of 9 (91%) samples were amplified successfully and finally for GT 3a 18 out of 19 (96%) samples were successfully amplified. Thus, in total there were 51 out of 54 samples (94%) sent for the population sequencing. Good sequence quality was defined by SeqScape® software as > 20% tolerance for improper sequencing. As can be seen in Table 2, A553V and C445F were a baseline polymorphism (i.e. found in 100% of samples) in GTs 2b and 3a . Two of the 5 GT 1b samples had C316N RAS, while 8 and 4 of the 8 GT 2b samples had C445F and S556G RASs, respectively. Eighteen out of 18 GT 3a samples had A553V and S556G RASs.

**Table 2.** RASs in HCV NS5B patient samples identified by the population sequencing method.

Resistance Polymorphism/Mutation	Genotype (total analyzed)								Fold resistance data for dasabuvir from literature	Nucleotide transition or transversion
	1a (n = 20)		1b (n = 5)		2b (n = 8)		3a (n = 18)			
	n	%	n	%	n	%	n	%		
C316N	0	0	2	40	0	0	0	0	5 (GT 1b) ^a^	Transistion/Transversion
C316Y	0	0	0	0	0	0	0	0	1472 (GT 1a) ^a,b^ and 1569 (GT 1b) ^a^	Transition
S368T	0	0	0	0	0	0	0	0	139 (GT 1b) ^a^	Transversion
A395G	0	0	0	0	0	0	0	0	10 (GT1a)^a^	Transversion
N411S	0	0	0	0	0	0	0	0	84 (GT 1b) ^a^	Transition
M414T	0	0	0	0	0	0	0	0	32 (GT 1a) ^a,b^ and 47 (GT 1b) ^a^	Transversion
C445F	0	0	0	0	8	100	18	100	16 (GT 1b) ^a^	Transversion
Y448C	0	0	0	0	0	0	0	0	940 (GT 1a) ^a^ and 414 (GT 1b) ^a^	Transition
Y448H	0	0	0	0	0	0	0	0	975 (GT 1a) ^a,b^ and 46 (GT 1b) ^a^	Transition
C451S	0	0	0	0	0	0	0	0	16 (GT 1b) ^a^	Transversion
A553T	0	0	0	0	0	0	0	0	152 (GT 1a) ^c^	Transition
A553V	0	0	0	0	8	100	18	100	120 (GT 1b) ^a^	Transition
G554S	0	0	0	0	0	0	0	0	198 (GT 1a) ^b^	Transition
S556G	0	0	0	0	4	50	18	100	30 (GT 1a) ^a,b^ and 11 (GT 1b) ^a,b^	Transition
S556N	0	0	0	0	0	0	0	0	29 (GT 1a) ^a^	Transition
S556R	0	0	0	0	0	0	0	0	261 (GT 1a) ^c^	Transversion
D559G	0	0	0	0	0	0	0	0	>100 (GT 1a) ^c^ and > 100 (GT 1b) ^c^	Transition
Y561H	0	0	0	0	0	0	0	0	20–100 (GT 1a)^c^	Transversion

References: a. Kati et. al. [20]; b. Lontok et. al. [24]; c. Sarrazin et al. [7].

### Modeling of the NS5B-dasabuvir complex

**Figure 1.**
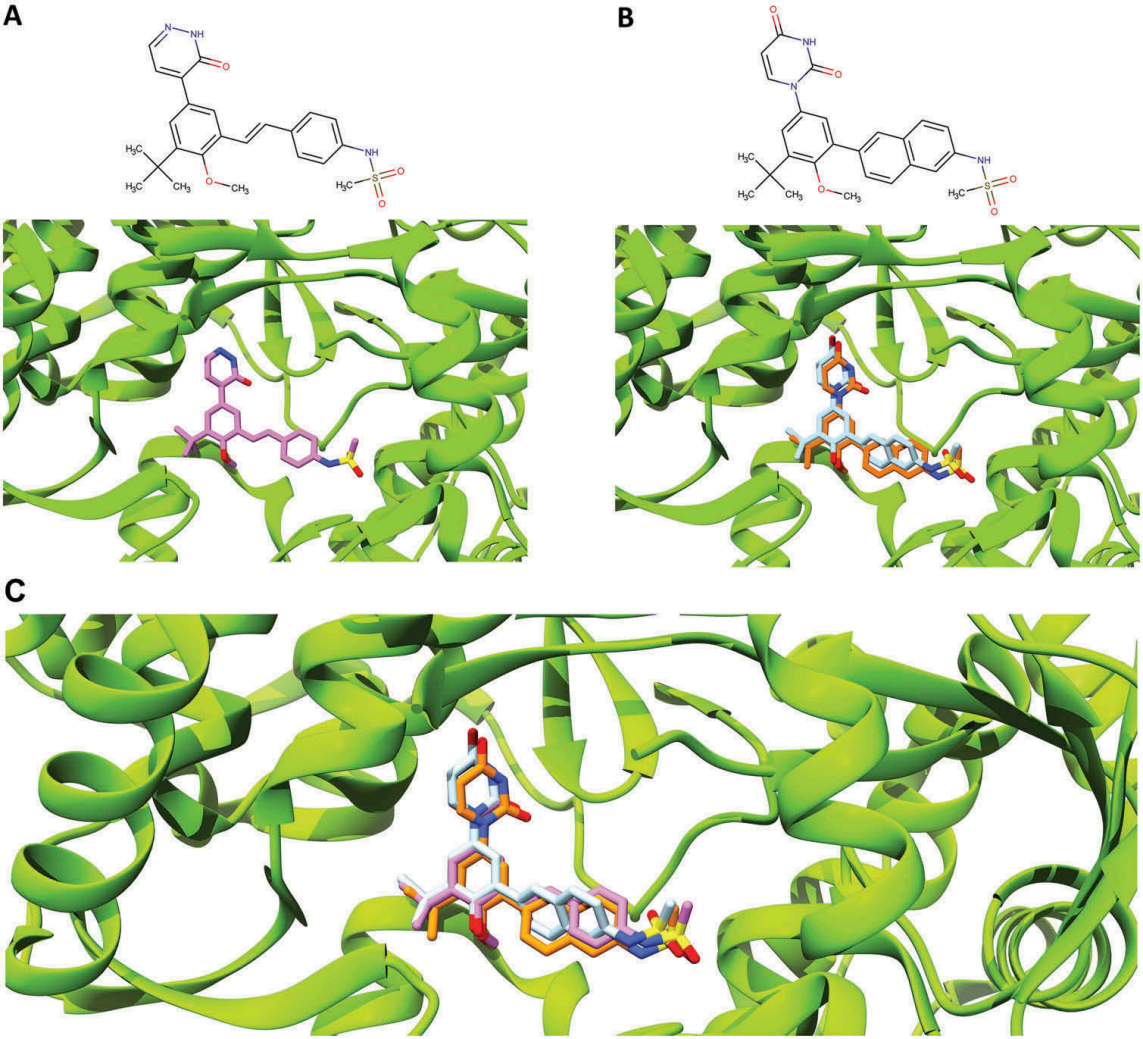
Comparison of the binding pose of compound 28V and dasabuvir. (A) The close-up view of the binding pose of 28V (purple) co-crystallized with HCV NS5B polymerase (PDB ID: 4MKB). (B) The close-up view of the binding poses of dasabuvir with HCV NS5B polymerase generated using AutoDock Vina (cyan) and iGEMCDOCK (orange). (C) The superimposed binding poses of compound 28V and dasabuvir.

A model or crystal structure of dasabuvir bound to HCV NS5B polymerase is not yet available. However, the pattern of resistance observed locates the dasabuvir binding site to the palm I of the NS5B polymerase [20,39]. In order to better understand and study dasabuvir RASs, we predicted the dasabuvir’s binding pose in complex with HCV NS5B polymerase using the *in silico* molecular docking and MD simulations’ methods. The HCV (GT 1b) NS5B polymerase, PDB ID: 4MKB [25], was used as a receptor to predict the dasabuvir’s binding pose. In the aforementioned PDB file, NS5B was co-crystallized with the compound ‘28V’ (PubChem ID 46220530) that is structurally related to dasabuvir (Figure 1(A,B)). Since the two compounds share a similar structure, we speculated that they might also share a similar binding pose and that the favorable conformation of the binding site would allow for a reliable predication of the dasabuvir binding pose.

Two programs namely, AutoDock Vina and iGEMDOCK were used for the molecular docking. The selection of the predicted correct dasabuvir binding pose was guided by the knowledge of the compound 28V’s binding pose and the binding pose reliability was further increased by choosing consensus results generated by both the docking programs. Therefore, from the results of each docking programs, consensus binding poses more similar to the compound 28V’s binding pose were considered correctly docked and were selected (Figure 1(A,B)). The agreement between the selected binding poses from both programs was evaluated by calculating the relative root-mean-square deviation (RMSD). The selected binding poses had a very low RMSD value of 0.63 Å, demonstrating that a consensus binding pose shared by both programs were successfully identified.

**Figure 2.**
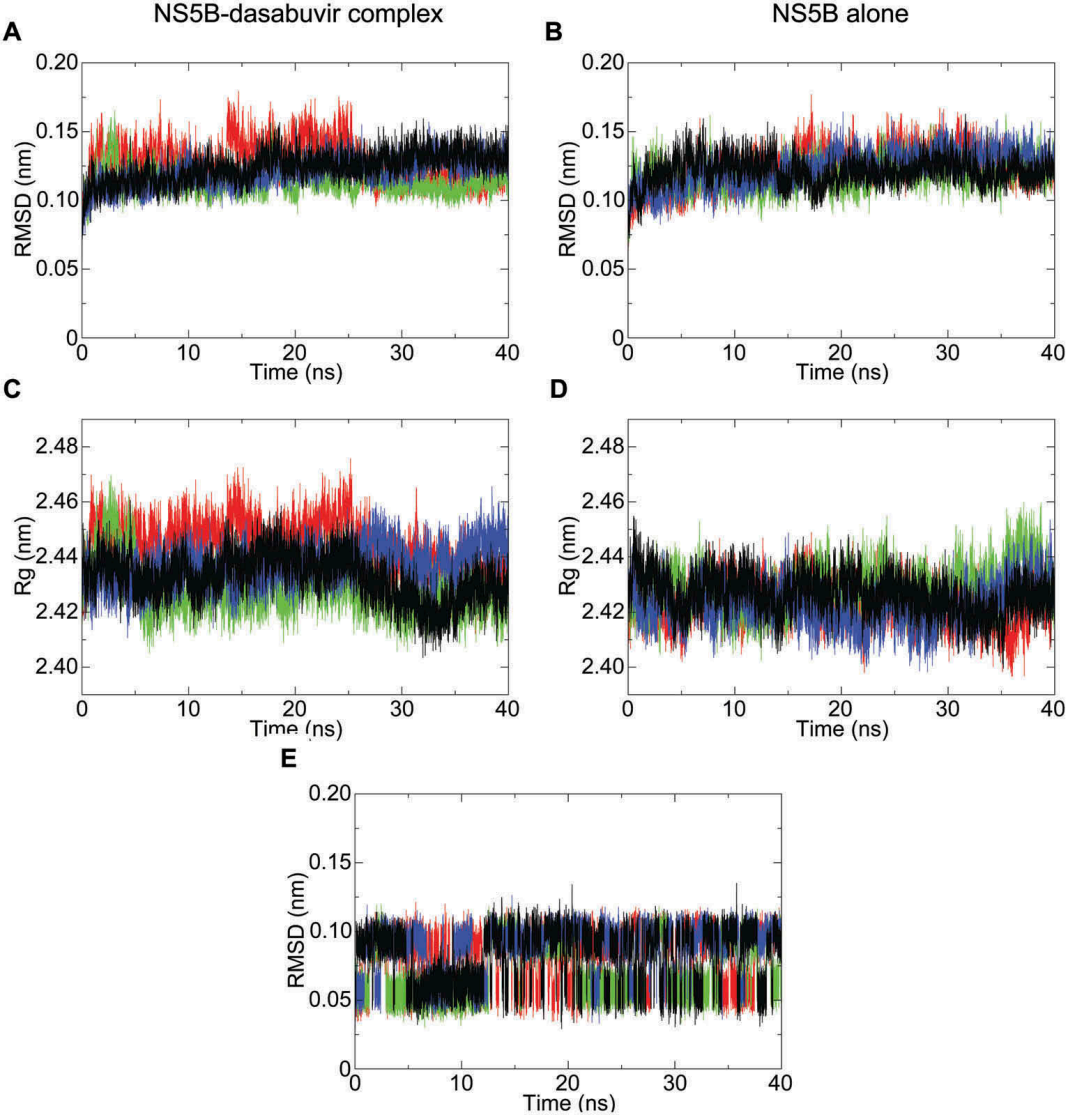
NS5B backbone root-mean-square deviation (RMSD) and radius of gyration (Rg) plotted with respect to time. The RMSD fluctuation of NS5B in complex with dasabuvir and of the free NS5B are shown in pictures (A,B), respectively. Similarly, the radius of gyration (Rg) of NS5B in complex with dasabuvir and the free NS5B are shown in pictures (C,D), respectively. In picture E, the RMSD fluctuation of dasabuvir over time is shown. The different simulation replicate is color coded as follows: the first simulation is shown in black, second simulation is shown in green, third simulation in red and fourth simulation in blue.

To investigate the NS5B-dasabuvir complex’s stability over time, the dasabuvir binding pose selected from the AutoDock Vina results was used as an input for the MD simulations. A total of 4 simulations of the NS5B-dasabuvir complex and NS5B alone, with each run of 40 ns, was performed using GROMACS. The stability of NS5B and NS5B-dasabuvir complex over time was determined by estimating the RMSD and the radius of gyration (Rg). As it can be seen in Figure 2(A,B), the RMSD of NS5B-dasabuvir complex and NS5B alone was comparable (i.e. lower than 0.2 nm). In particular, the average RMSD of both NS5B-dasabuvir complex and NS5B alone was 0.12 nm with a relative standard deviation of 0.012 nm and 0.011 nm, respectively. The Rg of NS5B-dasabuvir complex oscillated more during the simulations compared to the NS5B alone (Figure 2(C,D)), but the average values 2.43 nm (standard deviation = 0.01) and 2.42 nm (standard deviation = 0.007), respectively were almost identical further proving the high structural stability of NS5B independently from the presence or absence of dasabuvir. The conformational stability of dasabuvir was also assessed by calculating its RMSD during the whole simulation time. As it can be seen in Figure 2(D), dasabuvir had minimal structural variations during all the simulation replicas with values oscillating mainly between 0.05 and 0.1 nm and an average RMSD value of 0.08 nm (standard deviation = 0.02). Overall, these findings suggested that dasabuvir had no effects on NS5B’s structural stability and the binding pose generated by molecular docking was very stable during the entire simulation period. In order to identify the most representative NS5B-dasabuvir complex from the 4 simulation replicas, a cluster analysis of 1000 frames from the last 10 ns of each simulation trajectories were then performed. In each run, we found only one cluster of NS5B-dasabuvir complex conformation. This confirms that the NS5B-dasabuvir complex was very stable during the MD simulations as stated above. The most representative NS5B-dasabuvir conformation from each one of the four clusters was then saved and compared to each other by the RMSD calculation. The NS5B-dasabuvir complex structure, representative of the 4^th^ simulation’s cluster, was more similar to all the other analyzed structures from the remaining three clusters and was selected as the NS5B-dasabuvir complex with correct binding pose.

### Structure analysis of NS5B-dasabuvir complex and RASs implicated in dasabuvir resistance

**Figure 3.**
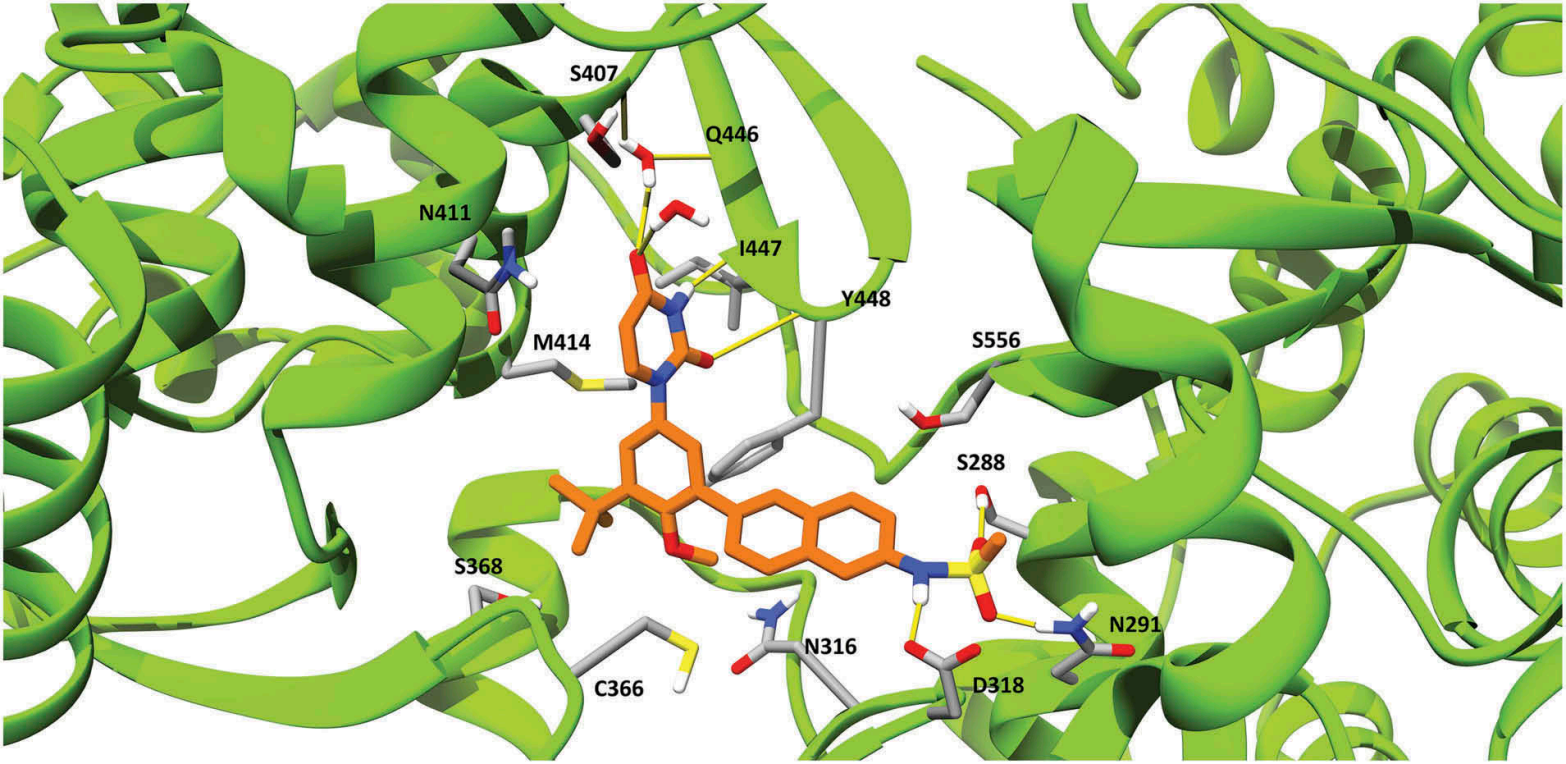
Dasabuvir’s binding pose with HCV NS5B polymerase generated using MD simulations. Dasabuvir is shown in orange, while HCV NS5B polymerase is shown in green. Clinically relevant HCV NS5B RASs and residues forming hydrogen bonds with dasabuvir are displayed with residue indexes. Hydrogen bonds are shown as yellow lines.

Ideal dasabuvir’s binding pose with HCV NS5B polymerase predicted in our study is shown in Figure 3. In this model, dasabuvir extends from the β-hairpin loop, formed by the residues L443 to I454 of the thumb domain, towards the NS5B active site. In particular, dasabuvir formed hydrogen bonds with the side chains of residues 288, 291 and 318 located close to the active site and with the backbone of residues 447 and 448 of the β-hairpin loop. This β-hairpin loop blocks the double stranded primer-template’s exit and it has to undergo an extensive conformational change to allow the elongation process [40]. However, dasabuvir might prevent such conformational change by interacting with residues 447 and 448 and overall stabilizing the β-hairpin loop. This is in accordance with the hypothesized general mode of action for the palm I binding non-nucleoside inhibitors, which are thought to bind and stabilize this β-hairpin loop thereby keeping the viral polymerase in an auto-inhibitory conformation [41]. In Figure 3, it can also be observed that a molecule of water makes a hydrogen bond with dasabuvir and residue 446 possibly further contributing to the stabilization of the β-hairpin loop.

**Figure 4.**
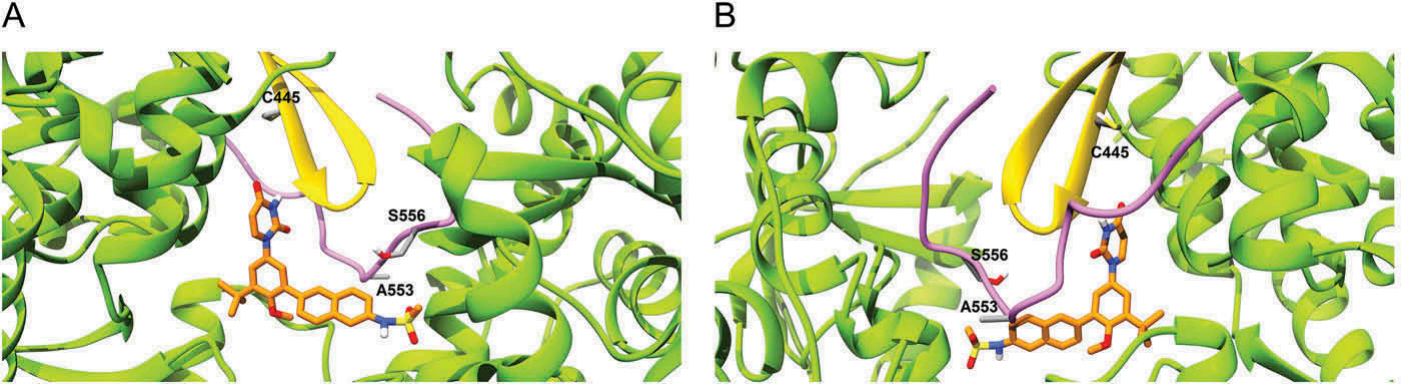
Location of residues 445, 553 and 556 in the dasabuvir binding site. (a) Front view and (b) Back view. In the picture, the β-hairpin loop is colored in yellow and the C-terminal tail in purple. As it can be seen, both the β-hairpin loop and C-terminal domain form a predominant part of the dasabuvir (colored in orange) binding site.

Mutation at residue positions 316, 414, 448 and 556 are associated with resistance to dasabuvir in both GTs 1a and 1b. In our model, these residues were found to be involved in shaping the dasabuvir binding pocket. In particular, the backbone of residue Y448 forms a hydrogen bond with dasabuvir while the other residues have their side chains facing the binding site and might be involved in van der Waals and other types of non-covalent interactions with dasabuvir. Dasabuvir also forms hydrogen bonds with the residues 288, 291 and 318. However, these residues are more conserved as previously shown and D318 is one of the residues constituting the NS5B polymerase’s active site [42]. Moreover, the importance of the residue position 291 was demonstrated by Ishii et al. which found that by introducing the mutations N291A and T287A in recombinant NS5B completely abolished the polymerase activity [43]. In our study, we found a high prevalence of variants C445F, A553V and S556G in both GTs 2b and 3a. The precise locations of C445 in the thumb β-hairpin loop, and A553 and S556 that are found in the NS5B C-terminal tail are shown in Figure 4.

## Discussion

It is well-known that the outgrowth of RASs during treatment failure can depend on negative factors such as baseline RASs with high-fold resistance towards DAAs. However, the general consensus is to recommend a cut-off level of 10 – 20%, for detecting RASs within the HCV quasispecies, in order to be of clinical relevance in predicting the viral failures [7,24]. Thus, the population sequencing method (or the next generation sequencing method set at a cut-off level of 10 – 20%) is thought to be the optimal method. In this study, we investigated the presence of baseline RASs in patient samples against the NNI dasabuvir by the population sequencing method. The population sequencing method was carried out on 54 treatment-naïve samples (GTs 1a, 1b, 2b and 3a) adopting the common cut-off level of ~20% for RASs detection.

There are only a handful of studies claiming to have designed HCV pan-genotypic primers covering all the DAA resistance sites in either NS3 protease or NS5A or NS5B regions [44]. As already pointed out in the Bartlett et al. review article [44], we have shown here that our designed primers are semi-pan-genotypic for the most prevalent HCV GTs globally (i.e. 1a, 1b, 2b and 3a), and covers the key NNI-DAA resistance sites in the NS5B region. It should be noted that the second set of primers designed by us (used for the nested PCR in this study) has been earlier reported in the review article [44]. Yet, the complete primers’ list has been unveiled only in this study.

By the population sequencing method, no RAS was found in GT 1a samples while the RAS C316N was found in 40% of GT 1b samples. The RAS C316N represents a baseline polymorphism of GT 1b (10.9 – 35.6%) associated with low level of resistance against dasabuvir (5-fold increase in effective concentration_50_) [7,20]. High prevalence of variants C445F, A553V and S556G were found in both GTs 2b and 3a. The variants C445F and S556G have also been previously reported as baseline polymorphisms with natural prevalence ranging from 96 to 100% for GT 2 and from 97.1 to 100% for GT 3 [7,42,45,46]. Additionally, the natural prevalence of the variant A553V was reported to be 96% for GT 2 and 97.1% for GT 3[46]. The variant A553V is associated with a high level (> 100-fold increase in effective concentration_50_) of resistance against dasabuvir in GT 1b replicon assay [20]. On the other hand, C445F and S556G are associated with low level of resistance (16 and 11-folds increase in effective concentration_50_ for C445F and S556G mutations, respectively) against dasabuvir in GT 1b replicon assay [20]. The mutations C445F, A553V and S556G found as a baseline polymorphism for GTs 2 and 3, they are considered resistance mutations towards dasabuvir for GT 1b [7,20]. All these RASs are located in the dasabuvir binding site in the palm I region of the HCV NS5B polymerase. In particular, the residue 445 is located in the β-loop that extends from the NS5B thumb while the residues 553 and 556 are located in the NS5B C-terminal tail. The possible negative effect of mutations at sites 445 and 556 are more evident than at position 553. In fact, as it can be seen, residue C445 is in close proximity to residues 446 and 448 which directly interact with dasabuvir while the residue S556 side chain is oriented towards dasabuvir and it is involved in shaping the dasabuvir binding site. However, the A553V mutation confers higher level of resistance compared to the mutations at residue positions 445 and 556. The role of NS5B C-terminus tail in conferring NNI resistance has been studied previously [47]. In the study performed by Boyce et al. (2014), the recombinant NS5B constructs namely Δ39 (C-terminus ending at position 552), Δ47 (C-terminus ending at position 544) and Δ55 (C-terminus ending at position 536), had more than 100-fold inhibitory concentration_50_ (IC_50_) increase against the palm one protease inhibitor A-837093[47]. The RAS A553V could cause structural changes of NS5B C-terminal tail that ultimately could result in the variation of the β-hairpin loop position through the interaction formed between the β-hairpin loop itself and the NS5B C-terminal tail. Such repositioning of key elements of dasabuvir binding site could have important effects on dasabuvir’s binding affinity towards the palm I site of the NS5B.

The presence of the variants C445F, A553V and S556G as a baseline polymorphism in GTs 2 and 3 might explain why dasabuvir, in combination with ombitasvir and paritaprevir, is approved only for the treatment of GT 1 patients [11]. Since dasabuvir is used only to treat GT 1 patients, there is no information on the possible negative impact of these variants on the treatment of other HCV GTs. Nevertheless, it could be expected that the variants A553V and S556G in GTs 2 and 3 would cause the same negative effect on dasabuvir efficacy reported for GT 1 even if the extent of the impact is not easily predictable.

To conclude, we did not find any potential clinically relevant RAS against dasabuvir in GT 1a and 1b samples by the population sequencing method. It is worth pointing out, that RASs against dasabuvir have very little impact on the treatment of GT 1b patients as reported by clinical trials [9,21–23,48,49] and literature reporting real life treatment data [50]. The known RASs C445F, A553V and S556G were found as baseline polymorphisms in GT 2b and 3a samples. These residues belong to the NS5B’s β-hairpin loop and C-terminal tail which form a predominant part of the dasabuvir binding site and thereby essential for the NNI inhibitory activity. Mutation of these residues could alter the conformation of the β-hairpin loop and in turn the shape of the dasabuvir binding pocket thereby possibly making the drug ineffective against GTs 2b and 3a.
